# Supplementary material for: Are carbon emissions trading and green financial instruments synergistic? -Comprehensive quantitative research based on content analysis
Source: PLoS One. 2024 Mar 7;19(3):e0298601. doi: 10.1371/journal.pone.0298601 (PMC10919601; doi:10.1371/journal.pone.0298601)
Supplement: S1 Data — (ZIP) [file pone.0298601.s001.zip › Data/地级市Z_F工作报告.zip!/║┌┴·╜¡/┤≤╟∞╩╨/└┤╫╘╣1⁄2╓┌║┼í╛┬φ┐╦╩2╛▌═°í┐_2016.docx]

各位代表：

　　我代表市人民政府，向大会报告工作，请予审议，并请市政协委员提出意见。

　　一、2015年工作回顾

　　 去年以来，全球经济总需求收缩，原油等大宗商品价格波动，受资源约束大庆石油减产，我市经济出现历史上首次负增长，完成地区生产总值2983.5亿元，下降2.3%。面对经济下行和腐败案件的双重冲击，在省委、省政府和市委的坚强领导下，我们保持战略定力，勇于担当作为，按照市委修复政治生态环境、寻求经济“十个突破”的要求，立足近期保运行、保运转、保民生，着眼长远抓产业、抓改革、抓环境，城乡居民收入稳定增长，全民创业形成氛围，新的发展因素和增长潜能加快汇集。——努力稳定经济运行。深挖4个方面31项增长潜力，争取增量多增一块、减量少减一些，新生力量加快形成。通过调整结构、丰富业态，拓展一产和三产增长空间。按照全产业链思路，调整种植业结构，大力发展畜牧业，粮食产量107.6亿斤，果蔬等经济作物产量48.2亿斤，农副产品市场化销售量价双增，一产增加值增长3.7%。催生新业态、新商业模式，增加服务业有效供给，电商企业新增215家，交易额130亿元，旅游收入达到96亿元，金融业进一步壮大，信息、物流产业加快发展，三产增加值增长6%。解决行业和企业发展个性化困难，遏制地方工业下滑幅度。支持停产半停产企业释放有效产能、有上升空间企业扩能升级、新投产企业上产达效。争取国家和省里政策性支持资金6492万元，开展银企对接协调贷款196.3亿元，7家企业在新三板成功挂牌。助力重点行业开拓市场，在大庆油田公司支持下，60家地方装备企业进入油田采购网。坚持多措并举，优化产业成长环境。制定建设国家现代农业示范区、发展电子商务、优化发展环境、推动创业创新等政策性意见和方案19个、措施300多条。推进简政放权，下调供热价格、非民用天然气和用水价格，解决企业审批难、建设难、融资难和生产经营成本高等现实问题，力促实体经济轻装前行。

　　——谋划和建设产业项目。从培育多元产业体系着眼，从既有需求增长空间又有供给优势的产业领域发力。拓宽石化项目生成渠道。推进“以化补油”战略，中直石化大企业7万吨石油磺酸盐等项目投产，在省委、省政府支持下，石化公司千万吨炼油项目启动前期工作。地方国有企业与炼化公司合作建设的15万吨丙烯等项目建成投产，混合所有制经济发展迈出关键一步。民营石化项目签约落地，为石化产业注入了新动能。推进战略性龙头项目建设。我市参与沃尔沃并购的资金已经开始回笼，spa平台建设顺利，实现产值110亿元、税收3.1亿元，以沃尔沃整车项目为牵动，加快建设汽车零部件产业园，江森汽车座椅等项目建成投产。完善忠旺集团200万吨高精铝深加工项目建设条件，加快推进全面建设的各项准备工作。抢抓全国云计算产业区域性布局的机遇，引入华为东北数据云中心、国裕“创业云+”等应用项目，带动大数据、互联网+、现代商贸物流等关联产业快速成长。升级发展地方重点产业链项目。深化市校合作，加速科技成果转化。层层传导招商压力，加快产业项目落地。重点推进福瑞邦靶向药物产业化、伊利奶粉价值链升级、兴和蛋业扩大产能等项目，新建续建超千万元产业项目181个、完成投资453亿元，新增产值超500万元科技型企业104家，全市科技成果转化率达到14.3%。

——保障和改善民生。面对财政减收压力，全力保障工资支出、事业运转和城市运行，在社会救助、低保投入、老居住区改造等方面优先安排，民生支出占公共财政支出比例比上年提高3.9个百分点。城乡居民人均可支配收入分别为34407元和13202元，增长6.5%和6.1%。支持全民创业带动就业。投入专项扶持资金1.77亿元，撬动融资11.5亿元，减免税费2.3亿元，惠及小微企业和个体户5.47万户。建立创业基地、企业孵化、创客空间等载体70个，吸纳企业2000余家。在商事制度改革推动下，新增个体工商户2.68万户、私营企业5054户，分别增长23.7%和10.7%。全市新增创业就业人员6万人，城镇登记失业率4.1%。提高保障水平。增加机关事业单位工资，人均月提高300元。城乡居民基础养老金最低标准提高到每月70元，企业退休人员养老金人均月增210元。新农合人均筹资标准提高到470元，城乡居民大病医疗保险最高支付限额达到20万元。城乡居民低保标准分别提高8%和27%，农村五保户集中供养、分散供养标准分别提高15%和13%。困难群体用电用热用气补贴、司法救助等市本级财政支出6400万元。支持61所医疗机构与93家养老机构签定“医养结合”合作协议，全市养老机构新增床位1100张。促进公共服务均等化。新建改建公办幼儿园和普惠性民办幼儿园32所、寄宿制学校和农村薄弱学校128所，远程教育共享教室达到400个，城乡义务教育标准化达标率提高到79.9%。新组建医联体20个，城乡人均基本公共卫生服务经费提高到40元，县级公立医院全部取消药品加成，外埠名院专家来庆诊治患者3.7万人次。建设中心村文体广场和农村文化大院60个，开展城乡文化惠民活动247场次，荣获省级以上奖项文艺作品82项。改善人居环境薄弱环节。启动“美丽乡村”建设，修复升级农村道路177公里，改造农村泥草房1.3万户。完成图强小区等老居住区改造，惠及居民5000户。加强铁路东西两站等重点区域治理，缓解居民小区和商业街区停车压力，新增停车泊位4万余个。改造供热系统，受益面积700万平方米。新建棚改安置房和公租房1629套。淘汰燃煤小锅炉272台、黄标车和老旧车2.4万辆。保持社会和谐稳定。全域开展安全生产大检查，打击非法违法行为2168起，安全生产形势稳定。落实信访案件领导包保机制，化解信访积案138件。社会治安防控体系进一步完善，八类主要刑事案件下降4.6%，一般火灾下降18%，交通事故亡人下降6.5%。

　　——推进改革开放。简政放权、放管结合、优化服务。再次取消、下放和调整市级行政审批事项112项，公布了政府权力清单和责任清单，减少合并办事环节113个，开通网上政务服务中心，审批事项全部纳入网上审批。理顺高新区和经开区管理体制，剥离社会职能，聚力产业发展。提高公共资产运营、公共资源配置和公共资金使用效率。市属国资国企改革破题，批准了市供热集团和市水务集团整合方案。清理市直行政事业单位非办公类资产，挂牌出售或市场化经营处置8处6万平方米，用于发展电子商务、健康养老等产业。农村土地承包经营权确权登记颁证工作全面实施。理顺历史形成的“一局两中心”多层次教育管理体制，强化区级职能，优化整合教育资源。针对市妇女儿童医院体制难题和市属三级医院发展不平衡等问题，在不增加机构和人员的前提下，成立大庆市医院联盟理事会，促进医疗资源共享。牢固树立政府过紧日子思想，市本级“三公”经费等一般性支出下降8.4%，盘活账面存量资金3.9亿元，清缴欠税欠费13.8亿元，通过强化财政资金使用审计和政府采购管理节支3.6亿元。积极扩大对外经贸合作。借助港粤招商活动周、中日韩企业对接会、第二届中俄博览会等平台，对接招商线索315个，引进项目168个，其中超亿元59个。农副产品销售、石油工程技术服务等对俄贸易取得新进展。

　过去一年，按照市委的要求部署，扎实开展“三严三实”专题教育，以“严”的精神、“实”的作风，强化基础性工作，提高专业化水准，政府依法行政能力和服务水平有新提升。严格执行市人大及其常委会的各项决议决定，自觉接受人大法律监督、工作监督和政协民主监督。市政府承办的223件市人大代表建议、308件市政协委员提案全部办结。

　　过去五年，城市面貌明显变化，交通功能、基础设施功能和大型场馆功能加快提升；社会事业取得进步，社会保障体系更加健全，人民生活质量稳步提高，城乡居民人均可支配收入年均分别增长10%和13%；改革开放不断深化，行政审批制度、医药卫生体制、基层社区管理、农业农村等重点领域改革扎实推进，对外经贸合作进一步拓展。主要受单一产业结构和原油价格影响，“十二五”期间经济发展波动较大，地区生产总值、地方财政收入年均分别增长6.1%和6.6%。

　　各位代表，我市经济社会发展进展来之不易。这是我们认真贯彻党中央、国务院和省委、省政府方针政策的结果，是市委统揽全局、坚强领导的结果，是石油石化大企业、驻庆高校等中省直单位合力支撑的结果，是全市人民团结奋斗的结果。在此，我代表市政府向各位代表、各位委员和全市人民，向所有关心支持大庆发展的同志们、朋友们，表示崇高的敬意和衷心的感谢！

　　回顾一年来的政府工作，除了资源性矛盾客观存在外，我们在破解难题抓发展上还存在一些差距和不足。一是结构性矛盾突出。三次产业结构在总量减少下出现了变动，一产和三产占比上升，三次产业比重达到6.5:65.4:28.1，但仍然没有摆脱结构失衡状态。工业一枝独大、占64.7%，规上工业中油化产业处于绝对支配地位、占80.5%，而非油比重仅为32.2%，产业链和价值链总体处于中低端。二是体制性矛盾明显。市场主体缺乏活力，资源配置市场化程度不高，地方国有企业发展动力不足，尤其是民营经济发展缓慢，非公经济只占23.1%，远低于全国全省平均水平。三是区域发展和民生水平不均衡。城乡之间、城区之间发展差距较大，县域经济总量不足全市的20%。在民生事业发展上农村存在很多短板，整体不均衡问题仍然比较明显，还有2个贫困县、90个贫困村、11万贫困人口亟待脱贫。四是政府工作与发展要求不适应。思想观念上不适应市场经济，想问题办事情还习惯于按计划经济和行政思维方式。工作状态上不敢担当、束手束脚，不作为乱作为等现象不同程度存在。能力水平上抓政府投资项目、办有钱的事比较擅长，抓产业培育、资产运营、社会融资等方面专业化水准比较欠缺。实际操作上不按规则行事，一些历史遗留问题亟待解决，一些“土政策”需要清理。这些问题我们要认真对待并切实加以解决。

二、“十三五”时期发展思路和重点任务

　　 今后五年是大庆转型发展的战略突破期。市委八届七次全会明确了“十三五”时期全市经济社会发展思路，总的目标就是与全国、全省同步全面建成小康社会，建成产业多元发展的经济大市、彰显大庆特色的文化名市、促进区域繁荣的中心城市、环境和谐优美的生态园林城市、人民群众认同感自豪感获得感不断提高的幸福城市。根本任务就是培育壮大接续产业，加快推动转型发展、可持续发展。主要遵循就是贯彻实施好创新、协调、绿色、开放、共享发展。到2020年，大庆油田油气当量保持在4000万吨规模，非油经济总量、地方经济总量、城乡居民人均收入比2010年翻一番。

　　（一）“十三五”时期面临的内外部环境。在经济增长放缓的同时，世界经济将经历深度调整，全球大宗商品价格从泡沫化状态回归常态。全国经济发展进入新常态，经济增速换挡、结构调整阵痛、动能转换困难相互交织，在总需求的有限空间内，结构性产能过剩比较严重，区域竞争愈发激烈。大庆原油减产，以石油为主体的工业经济难以大幅跃升，全市经济止跌回升、进入中高速增长区间的压力很大。同时，我们也面临难得的有利条件和发展机遇。一是大庆处在全省经济密度最高的哈大齐工业走廊中间位置，也是连接吉黑和绥满重要通道的组成部分，具备融入更大区域经济发展的良好地理区位。二是地下石油、天然气、地热，地上风能、太阳能、生物质能、耕地、草原、湿地、江河、湖泊等资源富集，有利于关联催生新的产业形态。三是原油产量梯度递减但仍处于较高水平，加之作为我国进口俄油俄气的集散地，能够为发展接续产业提供宝贵的掩护期和回旋空间。四是市政基础设施功能比较完善，教育、医疗、文化、科技等发展水平相对较高，具备吸引集聚更多人流物流信息流的承载环境。五是大庆精神、铁人精神传承弘扬，为我们应对挑战、克服困难提供了不竭动力。六是国家大力支持东北老工业基地振兴和资源型城市可持续发展，全省深入实施“五大规划”、加快建设“龙江丝路带”，我们在重大基础设施建设、重点产业培育、创业创新发展、改善保障民生、生态恢复治理等方面有更多更好发展契机。

　　（二）“十三五”时期发展的主攻方向。一是加快构建产业新体系，基本形成产业多样性发展格局。在服务油田产能建设的同时，推动石化、汽车和铝材等主营业务收入千亿元产业链，食品加工、装备制造、战略新兴产业、现代服务业等主营业务收入500亿元重点行业。挖掘农业结构调整关联诱导潜力，推动作物种植与牧业养殖对接、过腹增值，丰富农牧产品与食品加工链对接、产业化增值，优质食品与市场需求对接、营销增值，稳步提升一产综合效益。挖掘工业多点支撑潜力，基本建成国家级石化产业基地，沃尔沃、忠旺如期投产达产并发挥产业龙头带动效应，战略新兴产业实现集群发展，巩固提升二产支撑作用。挖掘服务业融合发展潜力，构建以城市旅游、文化休闲、现代流通、现代金融、现代信息服务、健康养老为总体框架的服务业新格局，加快提升三产贡献率。二是增强民生发展的整体性和均衡性，普遍提高人民生活水平。调整民生工作思路，民生大事实事的决策阶段充分听取群众意见，实施过程广泛吸纳群众参与，结果成效认真接受群众评价。转变民生工作思维，切实保证民生投入力度的同时，注重通过合理规划、科学管理、动员群众等途径，办更多让百姓贴心、暖心、舒心的实事。创新公共服务提供方式，扩大政府购买公共服务，加强与社会资本合作，提升公共服务共建能力和共享水平。坚守底线、突出重点、完善制度、引导预期，根据群众需求，每年安排一批民生实事，紧紧抓在手上，久久为功，确保教育、医疗、文化、养老、社会保障等公共服务均等化水平明显提高，确保贫困县和贫困人口全部脱贫，确保人民群众精神和物质上的获得感切实增强。三是强化功能环境系统治理，城市更加宜居宜业。坚持框定总量、盘活存量、做优增量、提高质量，完善城市治理体系，建设和谐宜居、富有活力、独具特色的现代化城市。巩固提升国家环保模范城市和卫生城市，积极创建全国文明城市。加快推进城区老工业区搬迁改造，“腾笼换鸟”发展现代服务业。统筹新区开发、老居住区和城中村改造，兼顾地上地下发展，提升现代立体交通体系，增强城市基础设施功能，做好生态修复和土壤、水体、大气环境等治理，提高智能化、精细化、市场化管理水平，强化城市系统性、持续性、宜居性。四是深化改革扩大开放，结构性和体制性调整取得实质进展。抓住国家供给侧结构性改革重大机遇，推动大众创业、万众创新，加快培育形成更具竞争力的市场供给主体；推动公共资产资源配置市场化，通过合并重组、混合发展、退出竞争领域等措施深化市属国有企业改革，提高国资国企经营效益；推动教育体制、医疗体制、文化体制改革，提高公共服务优质均衡供给水平。对接国家“一带一路”战略，拓展对外物流大通道，培育跨境跨地区产业链，使大庆融入更大区域经济循环。

（三）“十三五”时期抓产业项目生成的主要路径。一是向要素引入大力招商要项目和增长点。突出重点产业、重点园区，建立完善集项目谋划生成、专业力量推进、政策保障支持、产业基金引导、考核评价激励于一体的招商引资工作机制。探索产业链招商、地企携手招商、校地联合招商等新模式，促进市域内平台共用、人才共引、项目共建、利益共享，争取在引进战略投资者上取得更大突破。二是向资源开发精深加工要项目和增长点。依托丰富的石化、粮食、果蔬、农畜产品等资源，走精深加工路子，实现更多“全产业链”发展。深度开发“原字号”，支持重点企业技术改造和设备更新，开发适销对路新产品，提高产品的科技含量和附加值，全力把资源优势转化为经济优势。三是向存量扩张挖潜要项目和增长点。通过数量裂变式、合资合作式、资产重组式等路径，盘活做大现有经济存量。支持中小微企业成长壮大、成熟企业改造升级、投产企业达产达效，盘活有市场前景的困难企业，力争做大做强一批本土优势企业。四是向混合所有制经济突破发展要项目和增长点。引导支持地方国企、民营企业与中直大企业合作，共同培育“央企实力+地方活力”的混合所有制企业。重点围绕油气资源开发、石化原料吃配、石油装备制造、工程技术服务等优势领域开展嫁接合作，力争建设一批大项目，培育一批竞争力强的上市企业。五是向高新技术成果产业化要项目和增长点。促进校地校企深度合作，充分释放大专院校、科研院所创新潜能，深入实施三百科技型企业三年行动计划，打通科技成果梳理、成立公司、平台孵化、与资本市场合作等关键环节，推动战略新兴和高新技术产业集群发展。六是向新业态新商业模式要项目和增长点。把握产业发展信息化、专业化以及跨界嵌入特征，丰富新业态，引育新模式，推动产业迈向中高端。促进互联网与各产业形态、组织形式广泛深入融合，拓展众创、众包、众扶、众筹空间，力促创意生成项目、项目催生企业、企业托起产业。七是向政策落地变现要项目和增长点。围绕国家支持东北地区建设经济支撑带、先进装备制造业基地和重大技术装备战略基地、国家新型原材料基地、现代农业生产基地和重要技术创新与研发基地等重大决策，逐项对接谋划项目、争取支持，培育一批具有战略性的新经济增长点。

三、2016年工作安排

今年是“十三五”规划开局之年，是全面建成小康社会进入决胜阶段的第一年。政府工作总体要求是：全面贯彻党的十八大、十八届三中、四中、五中全会和中央经济工作会议，省委十一届六次全会和省委经济工作会议精神，深化落实市委八届七次全会决策部署，坚持创新、协调、绿色、开放、共享的发展理念，紧紧围绕全面建成小康社会目标，紧紧扭住培育壮大接续产业这一核心要务，着力增动能蓄潜能、稳增长调结构、惠民生促创业，努力在破解结构性、资源性、体制性矛盾，解决不平衡、不协调、不可持续问题上取得新进展，实现“十三五”时期经济社会持续健康发展的良好开局。综合考虑各方面因素，全市地区生产总值预期增长1%左右，非油经济增长4.5%，地方经济增长6%，城乡居民人均可支配收入分别增长7%和5%。

（一）多点增添动能，推动创新发展。抓改革、抓创业、抓环境、抓招商，提高市域内外、体制内外的要素资源利用效率。推动供给侧结构性改革增动能。支持科研人员、大学生和农民开展技术创业、创意创业、投资创业、互联网创业，引导企业转型“二次创业”，培育一批新的供给侧主体。加强产业园区功能建设投入，创新园区和大用户电力、天然气等能源供给方式，调整土地等级，精简归并“五险一金”收缴，推动融资合作和产业基金发展，建立用地“规划圈一次性审批”制度，有效保障企业发展要素资源，降低交易成本。依据法律法规和政策，清理处置长期占而不建、建而不投以及名存实亡企业的闲置资产，支持通过市场化途径实现有效利用。深化地企、市校融合增动能。合力落实省政府与中石油集团战略合作框架协议，建立健全地企常态化多层次沟通协调机制；共建经开区，联手建设油化装备制造业基地；创新合作模式，加大油气资源共同开发力度；统筹城乡建设规划和油田产能规划，搞好市政设施建设运营和生态环境保护。推动地方重点产业与高校资源深度嫁接，落实好市校150个具体合作项目；引进中科院长春分院、哈工大新能源和化工等创新平台，市校企共建研发中心或工程技术创新平台10个以上；实施好三百科技型企业三年行动计划，新增产值超500万元科技型企业100家以上。破除体制机制障碍增动能。继续大力度精简行政审批事项，依法取消行政审批中介服务，推动部门分设办事窗口整合为综合窗口，促进各级政府间、办事部门间信息互通共享，变“多头受理”为“一个窗口受理”、变“群众来回跑”为“部门协同办”。推进网上政务服务向县区、乡镇和社区延伸，构建“一个网络+三级平台+四张清单”的政务服务新格局。完成市属供热、供排水等国企改革，促进国有资产资本化，扩大市场合作机会。推动奥林匹克公园等大型文体场馆市场化专业化经营，持续清理处置非办公类闲置资产，重点向创造财富的生产部门配置。搞好龙凤湿地公园开发区域、国际会议中心等项目招商，尽快建成运营。全面实施教育管理体制改革，稳妥做好有关学校划转和资源整合工作，探索实行学校维修改造、设备采购和物业管理社会化。推进财税管理体制改革，进一步理顺市、区财税体制，积极争取中央企业税收分享等政策。多领域扩大开放增动能。推动以对俄为重点的经贸合作加快升级，跟踪中石油国际事业成品油、漠大线原油增量等进出口大项目，争取新增进口俄油俄气在大庆报关结算，谋划建设旅游、跨境电商等对俄合作项目。加紧申建综合保税区，争取大庆机场列入国家口岸开放“十三五”规划。层层传导招商压力，争取引进超亿元项目100个以上，到位资金300亿元以上。

（二）突出项目建设，积蓄工业力量。实施工业强基工程，稳油气、强石化、壮地方，打好规上工业保卫战。支持中直石油石化企业发展。服务油田实施“四大工程”，通过致密动用等综合增产措施，力争生产原油3750万吨，开采天然气38亿立方米。合力争取国家在大庆建设原油储备基地。做好油气生产用地预审、环境评价和施工保障，加强油气输送管线安全整治，维护油田生产秩序。稳妥理顺企业脱离办社会职能，支持油化大企业降本增效。加快建设国家石化产业基地。坚持中直与地方两个板块共同发力、国有与民营两支力量融合并促，延伸石化产业链条。支持石化公司千万吨炼油、炼化公司30万吨C4烷基化、中蓝石化15万吨石脑油改质等项目建设，共同争取扩大中直石化企业炼量。地企联合组建市场主体发展混合所有制经济，建设120万吨甲醇制烯烃、20万吨醋酸装置改造等项目。推动民营石化重点项目建设，力促阿穆尔—黑河边境油品储运和炼化综合体下游、北京三聚环保百万吨重劣质油悬浮床加氢改质、安瑞佳汉阳科技C4综合利用等项目落地开工。围绕吃配15万吨乙烯、20万吨丙烯等基础化工原料，引进战略投资者，丰富下游产品线和价值链。利用石化产业优势，引入神雾集团建设煤质综合利用项目。壮大非油化支柱产业规模实力。启动实施重点工业项目集群建设行动计划，全年新建续建投资超千万元项目100个以上、建成投产40个以上。推进沃尔沃SPA平台建成量产，汽车零部件产业园投入使用，忠旺200万吨高精铝和120万吨板带箔项目开工建设。确保深层水平气井钻完井配套装置、兴和生物科技二期、福瑞邦脂微球载体制剂等项目投产达效，推动装备制造、食品加工、生物医药等产业扩容升级。建设砷化镓抛光片扩能、50兆瓦智能分布式光伏发电、量子点激光器、DCC装置再生烟气净化等重点项目，谋划地热能、太阳能、风能综合利用项目，打造国家新能源转型试点城市，带动新材料、新能源、新一代信息技术、节能环保等战略新兴产业发展。助力优质地方存量企业焕发生机。针对有效产能未充分释放和效益下滑企业实际，分类推进、量体裁衣、个性帮扶，扩能一批，转型一批，盘活一批，新增规上企业30家。

（三）提高综合效益，发展现代农业。坚持生态农业发展方向，推动农村一二三产业交叉融合，盯住市场需求变化促进结构调整，加快建设国家现代农业示范区。深化农村综合改革。全面完成农村土地确权登记任务，健全“五位一体”土地承包管理机制，为土地规模化经营创造更好条件。落实《推进农村产权制度改革实施意见》，重点做好经营性资产较大村改革试点工作。发展新型经营主体，加强合作社、家庭农场、专业大户和职业经理人队伍建设，新组建500万元规模的现代农机合作社10个以上。调整优化农业结构。立足全产业链发展优质粮食、安全牧业和绿色果蔬，推动农业结构往畜牧养殖、加工原料、市场需求上调，由种得好、养得好向卖得好转变，倒逼种得养得更好。扎实推进“三百园区”工程，通过节水增粮、松嫩两江旱改水等措施，新增高标准农田30万亩、水田30万亩，发展果蔬、杂豆、花生、汉麻等经济作物46万亩，引入国内外乳业、肉牛、生猪饲养和加工企业，改造提升“两牛一猪三禽”养殖场80个、新建单产8吨以上示范奶牛牧场30个。突出草原乳肉、黑土杂粮、两江水稻、寒地果蔬等优势产业链生成和企业引建，加快发展冷链物流、礼品农业、订制农业等新业态。健全涉农服务体系。实施“互联网+农业”行动计划，发挥国裕“创业云+”等农业物联网平台功能，启动29个示范基地，建设涉农产业生产溯源体系、标准安全体系和现代营销体系。引导银行等金融机构创新涉农金融产品，扩大农民合作社资金互助、农村合作金融公司、农业金融租赁公司试点范围。支持八一农大、省农科院大庆分院在良种繁育、农作物栽培、农产品加工等领域加大科研攻关和成果转化力度。

　　（四）丰富服务业态，促进三产升级。抓产业关联，抓业态融合，抓有效供给，积极构建现代服务业新格局。构建城市休闲服务体系，促进特色旅游、文化创意、生态资源组合开发。通过市场化运作办好湿地旅游文化节、雪地温泉节、文博会、斯诺克国锦赛、全国铁人三项赛等节庆会展和文体赛事。在歌剧院、石油馆及有条件的景区开办民族歌舞、精品舞剧、儿童剧、石油文化剧和经典音乐会等驻场演出，并贯穿旅游季。打造市域内全季节休闲、跨区域冬夏两季旅游的精品线路，推动旅游线上线下定向营销。谋划推进大庆休闲创意文化博览园、帆船帆板和龙舟训练基地、阿木塔蒙古族风情园、“鹤鸣海”湿地温泉度假区等项目建设。构建现代流通服务体系，促进传统商贸、电子商务、仓储物流线上线下互动。立足补短板、降成本、强功能，提升区域性大进大出流通链条。引导现有传统商圈向创意商圈、智慧商圈、休闲商圈转型。建设国际大宗商品交易中心，深化与黑河、满洲里等对俄口岸的报检通关合作。推动新商所、新交所增加交易品种，建好高新区和萨尔图区电商产业园、红岗大宗商品电商交易园，积极创建国家级电子商务进农村示范县，鼓励淘大庆等企业布局社区网超。加快建设龙凤农产品物流园、大同综合物流园，增强区域性集散功能。构建现代融资服务体系，促进银行信贷、证券市场、产业基金多业并举。完善银企对接机制，信贷投放力争超过1000亿元。设立产业引导基金，首期筹资40亿元。积极争取发行一般性地方政府债券和专项债券，继续引进金融机构，发展民营担保企业，引导银行保险机构开展互联网支付等新兴业务。支持企业对接新三板、战略新兴板等资本市场，力争新增上市挂牌企业10家以上。构建现代信息服务体系，促进基础设施、网络覆盖、数据应用一体推进。推动华为东北数据云中心投入运行，促进大数据、云计算、物联网技术手段与现代制造业、公共服务、政务服务紧密融合。实施光网城市建设工程，超前规划建设信息基础设施，3g/4g网络覆盖率、公共场所免费wifi覆盖率达到90%以上，提高网络高效不间断运行质量。构建健康养老服务体系，促进社会化养老、生态化养老、信息化养老协调发展。培育托管托养、康复护理、家政陪护、绿色配餐等“银发产业链”，支持厂矿医院利用闲置资源开设医养结合病房、有条件的民办养老机构完善医护功能。引导民间资本发展养老产业，建设“互联网+养老”服务项目，推进终端进社区进家庭。鼓励生态景区开发疗养休养功能，推动温泉养老花园等项目建设，发展旅游养老。（五）立足比较优势，壮大县域经济。坚持以推进新型城镇化为牵动、全市经济一盘棋，在产业规划、政策制定、资金投入、项目摆放等方面加大对县区支持力度，发挥县区主观能动性，提高县区经济贡献率。优化空间布局。落实城区老工业区搬迁改造方案，引导龙凤区、让胡路区和萨尔图区工业企业向专业园区集中，“退二进三”发展现代服务业。缩小城区之间功能落差，支持红岗区、大同区完善生产生活服务配套建设，增强综合承载能力。着眼产业差异化发展，推动四县做强特色主导产业，打造外围支撑板块。培育支柱产业。指导县区明晰产业定位，宜农牧强农牧、宜加工强加工、宜旅游强旅游、宜流通强流通，每个县区加快形成1—2个主导产业、每个产业培育1—2个龙头项目。引导县区把抓产业、上项目、扶企业、增税源作为重中之重，积极探索园区建设、人才引入、校地合作、“飞地”经济等新模式，确保县区经济增长速度不低于全市地方经济总量增速。提升基础功能。赋予县区在城镇管理、安全监督、环境保护、义务教育等方面更大的行政管理权限。支持县区依据城镇人口和产业发展趋势，通过PPP等模式加强道路交通、网络通信、教育医疗、文化服务和市政基础设施建设，促进优质公共服务向乡镇、社区、村屯特别是薄弱部位延伸。建好美丽乡村。深入实施美丽乡村“三年行动计划”，完善提升示范村50个、重点村196个、达标村237个。继续改造农村泥草房和危房，推进村庄绿化、屯内亮化、庭院美化，积极争取和建设农村垃圾污水集中治理项目，通过生物质发电、有机还田等措施搞好秸秆综合利用。

（六）注重民意导向，着力改善民生。从增强整体性、均衡性和精准性切入，结合广泛征求的民生工作意见建议，着重抓好9个方面41件民生实事。一是扶贫脱贫方面。落实县级主体责任，调动各方积极性，形成政府真抓、干部真帮、群众真干的局面。(1)积极向上争取扶贫脱贫专项政策资金。(2)对有资源优势的村屯实施开发式扶贫，实现整体脱贫。(3)通过技能培训、转移就业、扶持创业等办法帮助基础条件差、发展能力弱的贫困户增收。(4)将因病致贫、丧失劳动能力的贫困人口纳入低保、综合救助，帮助改善生活质量。二是全民创业方面。坚持政策集成促动，多元主体带动，综合承载平台牵动，持续激励全民创业，扶持创业实体2.5万个、带动就业6万人，城镇登记失业率控制在4.5%以内。(5)充实专项扶持资金。(6)完善税费减免、股权合作、众筹融资、创业指导团队建设等支持政策。(7)提升大学科技园、中科创业园、新型产业孵化器等平台，鼓励企业和民间资本自建创业平台，吸引各类创业要素集中集聚、有效对接。(8)支持中小微企业、“瞪羚企业”发展。三是社会保障方面。(9)落实社会保险关系转移接续政策，推动油化企业社会保险属地管理。(10)加快机关事业养老保险并轨衔接。(11)推进城镇医疗保险市级统筹，推动省内异地就医即时结算。(12)按政策提高城乡最低生活保障标准、农村五保户供养标准和市区高龄老人生活津贴标准，及时救助因病致贫、因突发事件返贫等家庭。(13)健全老年人乘车、旅游、体检等优待政策，探索建立医养结合补贴政策和保险政策。四是教育事业方面。(14)通过购买服务、以奖代投等办法，加快标准化普惠性幼儿园建设。(15)优化教育资源配置，整合规模小、生源少等薄弱学校，确保5个区和杜尔伯特县达到义务教育均衡发展标准。(16)创新实习实训方式，推动职业教育与创业培训、重点产业技工人才培养紧密衔接。五是医疗卫生方面。(17)深化与国内国际名院合作，加快市县医联体建设，推进县乡村医疗卫生一体化。(18)加强基层医疗卫生服务能力建设，完善医疗设备，保障村卫生室运行经费。(19)实施乡村医生专业技术培训工程，通过购买服务方式开展学历教育和技能培训，扩大乡村职业助理医师比例。(20)实施全面二孩政策。六是文化体育方面。(21)提高现有各类体育场馆开放率和利用率，建设一批社区和行政村综合文化活动场所，发展一批基层文化团体。(22)加大政府购买公共文化服务力度，引导社会力量参与公益性文化活动。(23)办好千场文化惠民展演，鼓励开展冰雪体育运动、普及校园足球。七是城市功能方面。(24)建设萨尔图机场国际航站楼。(25)谋划推动哈大、大齐高速连接线，规划建设连接大广、大齐和大绥高速公路的城市外环高速，谋划经济合理方式建设联结东西城快速城市交通和轨道交通。(26)建设铁路西站地区西杨路、西柳街、科学路，修复热源街、兰德路等10余条破损道路和无灯路，加固广电桥、团结桥、西宾桥等桥梁。(27)完善各工业园区污水处理设施，通过PPP模式建设光明污水处理厂。(28)按照海绵城市建设标准，完善城市供排水系统，加强集雨型绿地和生态用水补给设施建设，改造城区14处易积水路段排水管网。(29)新建改建东城区供热一级主干网，完善热源联网体系。(30)加快地下管网普查，编制地下管廊建设管理专项规划。八是城市管理方面。推进城市执法体制改革，完善分级属地管理体制。做好国家卫生城市复检、全国文明城市复审工作。(31)建设全国信息惠民试点城市，建立数据资源共享、惠民融合服务、食药安全服务、创业就业服务、不动产登记、智慧旅游等12个平台，提高城市智能化综合管理水平。(32)整合用好物业、城管、公安的视频探头监控系统，提高覆盖率、完好率和综合利用率。(33)改造提升建安、筑安、利民苑等6个老旧小区环境，着力解决漏雨、低温、管网破损等问题。(34)科学规范城市干道、校园周边、商业街区、客运站场等重点部位行停车秩序，优化公交线路，严厉打击“黑车”。(35)在人车密集通行节点建设过街设施，增加居住小区停车泊位，完善城乡道路交通标识标线等设施。(36)划定中心城区高污染燃料禁燃区，改造集中供热燃煤锅炉环保设施，继续淘汰燃煤小锅炉和黄标车，启动青龙湖环境综合治理工程。(37)坚持疏堵结合，大力整治小广告，规范治理露天集贸市场和露天烧烤。九是社会治理方面。(38)加强企业生产、校园、交通、消防、地下管网等重点领域安全监管，对重大隐患实行最严格的整治措施，完善高层住宅和公共场所消防人防设施。(39)实施以促进绿色食品产业发展为核心的食品安全战略，健全食品安全追溯体系，建成食品药品检验检测中心。(40)立足事要解决，落实信访三级终结程序，切实解决群众合理诉求，依法处理各类信访问题。(41)完善“六张治安防控网”，做好特殊人群服务管理，严厉打击多发性侵财犯罪和非法集资等涉众型经济犯罪，全力维护人民群众生命财产安全。

　四、加强和改进政府工作

　　 面对“十三五”时期大庆可持续发展的新挑战，落实市委“促发展、惠民生、敢担当、有作为”的新要求，回应全市人民的新期待，坚持问题导向和目标导向，搞好政府自身建设。

　　（一）转变理念观念，提高政府工作专业化水平。顺应新趋势，掌握新政策、学习新知识、适应新变化，牢固树立创新、协调、绿色、开放、共享的发展理念，对不适应不适合甚至违背新发展理念的认识立即调整、行为坚决纠正、做法坚决摒弃。增强市场经济的思维观念，善于借助资本市场和运用市场化手段配置资源，提高公共资产运营、社会资本合作、商务谈判等方面的专业水准。关注和适应科技革命和产业变革，强化推动新技术、新产业、新业态、新商业模式的专业思维、专业素养和专业方法。注重利用大数据提升统计分析、行业研判、趋势性把握等咨询决策能力，政府及其部门从一把手到副职、到科室工作人员，做到政策清楚、数字清楚、情况清楚、问题清楚。

　　（二）尊法学法守法用法，提高依法行政水平。落实《法治政府建设实施纲要》，做好全省“建立法治政府建设指标体系”试点市工作，通过建设法治政府推动法治大庆建设。严格执行权力清单和责任清单，按照法定权限和程序依法行政。本着对人民负责、对事业负责、对发展负责，不遗余力地处理好城建、民生、改革等方面历史遗留问题。坚持依法依规办事，推广行政执法检查“两随机、一公开”，避免发生违法行政和不公正不文明执法现象，加强行政复议工作，努力将行政争议化解在行政程序中，减少行政诉讼案件。自觉接受党内监督、人大监督、民主监督、行政监督、司法监督、审计监督、社会监督和舆论监督，认真办理人大代表建议和政协委员提案，支持工会、共青团、妇联等群团组织开展工作。

（三）履职尽责，敢于担当，提高政府执行落实水平。强化全局意识和大局观念，紧紧围绕市委的中心工作，紧紧扭住培育壮大接续产业这一核心要务，强化招商引资、项目建设、“三上”企业发展、民生实事落实的通报考评，各级政府抓产业项目，主要领导亲自上手，对上争取亲自出马，招商对接亲自出面，层层传导发展压力。根据行政事权调整、机构职能重新界定、网上审批开通等新变化，创新服务方式，打通关节，提高效率。健全督查问责机制，严格督办查处不定事不担责、挂空档不走道、敷衍了事没工作含量等不作为慢作为的问题，对破坏发展环境的人和事公开曝光、倒查问责，对督查发现的问题和整改进展情况，及时向社会公布，使督查激励问责发挥出威力。进一步畅通政令、强化诚信，政府决策决定一旦做出，说了要算，定了要干，特别是在产业政策、招商承诺和民生实事的落实上，坚决避免“放空炮”，以诚信政府建设的新成效带动诚信社会建设实现新变化。

　　（四）严守纪律，完善制度，提高政府廉政建设水平。坚持把纪律和规矩挺在前面，严格贯彻《廉洁自律准则》和《纪律处分条例》，巩固提升“三严三实”专题教育成果，深入整改“四风”问题。牢固树立政府过紧日子思想，继续削减“三公”经费，提高公共资金资产资源管理效益，花好财政每一分钱。扩大财政资金预决算、国有资产运营、公共资源配置、重大建设项目批准和实施等领域的信息公开，整合政府采购、工程建设招投标、土地交易等内容建立公共资源交易中心，强化重点项目、重大投资、重要领域的行政监察和审计监督，构建科学规范、长效管用的廉政制度体系。

　　各位代表！新的一年，大庆踏上新的征程。我们相信，在全面建成小康社会的前进道路上，任何挑战都动摇不了我们开创美好未来的坚定信念，再多困难也阻挡不了全市人民奔向幸福生活的坚定步伐。只要我们在市委的坚强领导下，发扬大庆精神、铁人精神，攻坚克难，奋发有为，就一定能够取得大庆可持续发展的新胜利。
